# The Effect of Physicochemical Properties of Perfluoroalkylsilanes Solutions on Microtribological Features of Created Self-Assembled Monolayers

**DOI:** 10.3390/ma13153357

**Published:** 2020-07-29

**Authors:** Michał Cichomski, Ewelina Borkowska, Milena Prowizor, Damian Batory, Anna Jedrzejczak, Mariusz Dudek

**Affiliations:** 1 Department of Materials Technology and Chemistry, Faculty of Chemistry, University of Lodz, Pomorska 163, 90-236 Lodz, Poland; ewelina.bystrzycka@chemia.uni.lodz.pl (E.B.); milena.prowizor@chemia.uni.lodz.pl (M.P.); 2 Department of Vehicles and Fundamentals of Machine Design, Lodz University of Technology, Stefanowskiego 1/15, 90-924 Lodz, Poland; damian.batory@p.lodz.pl; 3 Institute of Materials Science and Engineering, Lodz University of Technology, Stefanowskiego 1/15, 90-924 Lodz, Poland; anna.jedrzejczak@p.lodz.pl (A.J.); mariusz.dudek@p.lodz.pl (M.D.)

**Keywords:** perfluoroalkylsilanes, viscosity, surface tension, self-assembled monolayers, thickness, microtribological properties

## Abstract

The presented article shows the influence of concentration of perfluoroalkylsilanes in solutions on tribological properties of self-assembled monolayers (SAMs) deposited on three surfaces with different silicon content in the millinewton load range. The SAMs were created using the liquid phase deposition (LPD) method with 1H, 1H, 2H, 2H-perfluorodecyltrichlorosilane (FDTS) and (3, 3, 3-trifluoropropyl) trichlorosilane (FPTS) solutions, for which viscosity and surface tension were estimated. Deposited layers were analyzed in terms of thickness, coverage, wettability, structure and coefficient of friction. The obtained results demonstrated that SAMs created on the silicon-incorporated diamond-like carbon (Si-DLC) coatings possess the best microtribological properties. Systems composed of perfluoroalkylsilane SAM structures deposited on Si-DLC coatings are highly promising candidates as material for microelectromechanical applications.

## 1. Introduction

In recent years, nanotechnology has become one of the most dynamic developing fields of modern science [1,2,3], which allows structures to be obtained at the nanometer scale and new materials to be obtained with specific properties and consequently application of those materials for the miniaturization of devices [3,4,5]. The construction of devices with contact surfaces of the order of micro- and even nanometers resulted in the need to search for new nanomaterials as well as substances to protect their surfaces. These substances should have excellent tribological properties, i.e., low values of adhesion forces and friction coefficient, as well as the ability to reduce surface wear. Self-assembled monolayers (SAMs) made of perfluoroalkylsilanes exhibit such properties. In addition, the choice of perfluoroalkylsilanes for surface modification is due to their ability to form a siloxane network by which the formed layer is well ordered and resistant against various environmental factors. The uniform surface coverage, without any defects, reduces the contact area between two elements of tribological system which results in lower friction and wear. As literature data indicate, SAMs of perfluoroalkylsilanes protect the surface against corrosion and ensure their long functionality.

Among the available methods of production of self-assembled monolayers (SAMs), the most effective is liquid phase deposition (LPD). These structures are organic molecular assemblies which spontaneously form dense and ordered layers with a thickness of single nanometers on appropriate substrates [6,7,8,9]. Depending on the molecular design, SAMs provide a way to tailor surface properties such as wetting [10,11] adhesion [12,13], friction [14,15,16] and antimicrobial [17] and corrosion properties [18,19,20]. Their high attraction is also driven by the simple preparation procedure, the reproducible film quality and stability of the monolayer. Therefore, SAMs are presently very interesting for scientists in the nanotechnology area [1,6]. So far, most of the fundamental studies have been performed with alkane [21], aromatic thiols [22] and dithiols [23]. Currently, more and more interest from the application point of view in microelectromechanical systems (MEMS) is focused on compounds containing silicon in their structure [24,25].

In organosilicon structures, the bonds between silicon and carbon compared to carbon-carbon bonds are longer (186 pm compared to 154 pm) and characterized by weaker bond dissociation energy (451 kJ/mol vs. 607 kJ/mol). Moreover, due to higher electronegativity of carbon in comparison to silicon, the C–C and Si–C bonds differ in polarization (for C–Si equal to 2.55 vs. for C–C equal to 1.90). The result of these properties are Si–O bonds in chemical reactions, which are much stronger compared to C–O bonds (809 kJ/mol versus 538 kJ/mol) [26]. Another positive effect of occurrence of energetically advantageous Si–O bonds is creation of perfluoroalkylsilanes network consisting of Si–O–Si linkages during the modification process. Additionally, surface modification by perfluoroalkylsilanes guarantees more satisfied physical-chemical properties than in the case of compounds that do not contain silicon in their structure [24,25,27]. 

Silicon plays a very important role in creating a self-assembled monolayer in the case of silicon-incorporated diamond-like carbon (Si-DLC) coatings. Our earlier studies [28,29] indicate that the more silicon that is in the DLC structure, the higher the affinity of fluoroalkylsilanes to the surface. What is more, using Si-DLC in MEMS/NEMS systems is beneficial since the addition of silicon to the DLC coatings changes their surface roughness and adhesion forces, reduces compressive stress and improves wear resistance. Furthermore, the use of silicon may delay the graphitization process and thus increase the durability of DLC at elevated temperatures [30,31].

Perfluoroalkylchlorosilanes are representative candidates of organosilicon compounds used as modifiers to improve mainly the tribological features. Organic layers built of spontaneously adsorbed molecules on investigated surfaces exhibited growing potential as protective agents, which can be used in reducing friction, adhesion and wear, which was confirmed by other research [32,33]. Obtained results indicate their application potential in electromechanical systems as components of sensors and actuators used in many fields of technology and medicine [34].

In the presented study, the primary destination was to reveal the influence of the concentration of two kinds of alkyl chain silane compounds with different lengths in toluene solvent on their viscosity and surface tension and finally on the microtribological properties of created SAMs. The modifier/surface systems were created with use of two kinds of compounds: 1H, 1H, 2H, 2H-perfluorodecyltrichlorosilane (FDTS) and (3, 3, 3-trifluoropropyl) trichlorosilane (FPTS). The modifications were performed on three surfaces: diamond-like carbon coating (DLC), silicon-incorporated diamond-like carbon coating (Si-DLC) and silicon. The surfaces were selected in such a way as to compare the influence of silicon content (the type of surfaces) on the effectiveness of modification.

For studies with Si-DLC coating/surface systems, coatings with high concentrations of silicon were selected. We also for the first time presented the use of viscosity and surface tension measurement results to optimize the concentration of modifier solutions proper for the modification.

The chemical composition of manufactured structures was obtained using Fourier transformed infrared spectroscopy (FTIR) analysis which allowed two important parameters to be determined: information about the coverage of the investigated surfaces and the thickness of the fabricated SAMs. The obtained thicknesses were confirmed using ellipsometry measurements. These parameters testified to the quality of the produced layers and allowed their physical-chemical properties to be determined. The ultrathin assembled monolayers produced using the LPD method were investigated in terms of wettability and tribological behavior in the millinewton load range. These tribological studies fill the gap between measurement results obtained in the nanoscale and macroscale region usually performed by other authors.

## 2. Materials and Methods 

### 2.1. Materials

As substrates for the creation of self-assembled monolayers, DLC and Si-DLC coatings deposited using the radio frequency plasma enhanced chemical vapor deposition (RF PECVD) method were selected. The coatings were synthesized on a single crystal p-type Si (100) wafer (Cemat Silicon S.A, Warsaw, Poland), which was also used as the model material. The RF PECVD process and physicochemical properties of carbon base coatings were described in detail in the publication of Jedrzejczak et al. [35]. For the investigations, DLC and Si-DLC coatings containing 32at.% of silicon, deposited at 600V negative substrate bias, were selected.

### 2.2. Pre-Treatment of Substrates

Initially, surfaces were cut into 1.0 × 1.0 cm^2^ substrates and cleaned with ethanol (analytical grade, POCH S.A., Gliwice, Poland) in an ultrasonic bath to remove organic contaminents. Afterwards, the investigated surfaces were exposed to oxygen radio frequency (RF) plasma (Zepto plasma system, 40 Hz, 100 W, Diener Electronic, Ebhausen, Germany, 15 min at 50 W). The high hydrophilic character of the substrates after the treatment using plasma indicates the formation of a significant amount of polar surface silanols important to manufacture the covalent attachment of modifiers to the investigated surfaces [36].

### 2.3. Preparation of Self-Assembled Perfluoroalkylsilane Layers

The self-assembled monolayers were created from silane compounds FPTS and FDTS, on three (Si, DLC, and Si-DLC) surfaces, using the liquid phase deposition (LPD) method. The compounds were purchased from ABCR, GmbH & Co. KG, Karlsruhe, Germany. The FPTS and FDTS solutions at five different concentrations, 0.02%, 0.05%, 0.10%, 0.20% and 0.50% (wt%), were prepared by dissolving the compounds in toluene at room temperature under ambient conditions. Due to differences in reactivity of both the perfluoroalkylsilanes, the modification was carried out in a time of 60 min for FDTS and 30 min for FPTS.

Prior to the creation of SAMs, the FPTS and FDTS solutions were characterized. The surface tension investigations were performed using the Du Noüy ring method using a KSV Sigma703D (KSV Instruments Ltd, Helsinki, Finland) at 25 °C. The force referred to as the wetted length acting on a ring as a result of the tension of the withdrawn liquid lamella when moving the ring from one phase to another is measured in this method. The reported values of the surface tension are averaged values of at least three measurements. The measured surface tension of the toluene used for the preparation of the perfluoroalkylsilanes solutions was *σ* = 28.11 mN/m.

The viscosity measurements were carried out using an Ostwald viscometer (Sibata, Nakane Soka, Japan) measuring the time for 1 mL of the prepared perfluoroalkylsilanes solution and toluene to flow through the capillary under the influence of gravity. The ratio of the average flow time of the investigated solutions and solvent from at least three measurements in relative viscosity are presented.

### 2.4. Characterization of Surfaces and Prepared Layers

The characterization of pure surfaces and deposited perfluoroalkylsilanes films was conducted using Fourier transformed infrared spectroscopy (FTIR) (Nicolet iS50 FTIR, Thermo Fisher Scientific, Waltham, MA, USA). The spectrophotometer with an ultra-high sensitive, low noise, linearized mercury-cadmium-telluride (MCT) detector equipped with VariGATR™ grazing angle ATR accessory dedicated for analysis of thin layers was applied. All spectra were recorded by collecting 128 scans (128 scans of the pure substrate as a background was recorded) at 8 cm^−1^ resolution, in the spectral range of 700–4000 cm^−1^ in the flow of dry air. Pure investigated substrates were used for background spectra. The obtained data was analyzed using OMNIC 9 software from Thermo Scientific.

The thickness of the manufactured layers was determined using variable-angle spectroscopic ellipsometry (VASE) with a J. A. Woollam V-VASE ellipsometer (J.A. Woollam Co, Lincoln, CA, USA). Data was collected in the wavelength range 260–1300 nm at a 70° angle of incidence. A Cauchy dispersion model with constant parameters of 1.45, 0.01 and 0.0 was used to calculate the thickness of ultra-thin perfluoroalkylsilane layers.

Hydrophobicity of the substrates was determined using the static contact angle measurements obtained at room temperature using a Drop Shape Analyzer (DSA25 Krűss Goniometr, Krűss, Hamburg, Germany). Average water contact angle (WCA) values were obtained from measurements at five different locations on the specimen surface. The values of contact angles for water, diiodomethane and glycerin were determined by the use of the Advance program. On the basis of these investigations, surface free energy (SFE) and its components were calculated using the van Oss Chaudhury-Good method [37].

The friction tests of the investigated surfaces were determined with the use of a ball-on-flat tribometer (T-23, ITEE, Radom, Poland), working in the millinewton load range. The coefficient of friction is the average value obtained by measuring the friction force as a function of the normal load curve. A silicon nitride (Si_3_N_4_) ball, 5 mm in diameter, was used as a counterbody. The measurements were performed in technical dry friction conditions and repeated three times in three different locations on the sample surface. All specimens and counterbody movements as well as the load applying mechanism system were computer-controlled using Lab-View software. The applied normal loads were in the range of 30–80 mN. The sliding speed was 25 mm min^−1^ over the distance of 5 mm. The topography of the wear tracks after the tribological tests was analyzed using scanning electron microscopy (SEM) (Nova NanoSEM 450, FEI, Hillsboro, WA, USA).

The root mean square (RMS) of the surface roughness of samples was investigated using atomic force microscopy (AFM-Solver P47) (NT-MDT, Moscow, Russia) operating in an air-conditioned environment at RT and a relative humidity of 40 ± 5%. The RMS of the surfaces was obtained from the scan area 2 µm × 2 µm.

## 3. Results and Discussion

### 3.1. Effect of Physicochemical Properties of Solutions of Perfluoroalkylsilanes on Wetting Properties of SAMs

**Figure 1:**
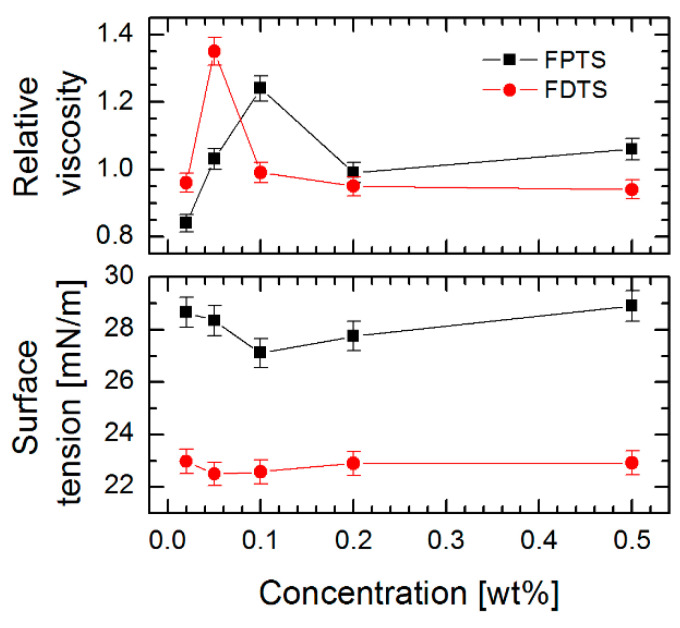
Influence of concentration of (3, 3, 3-trifluoropropyl) trichlorosilane (FPTS) and 1H, 1H, 2H, 2H-perfluorodecyltrichlorosilane (FDTS) compound on viscosity and surface tension of solutions.

The creation of SAMs using the LPD technique was preceded by the viscosity and surface tension measurements of the solution of two perfluoroalkylsilane compounds with different lengths of alkyl chain as a function of their concentrations (Figure 1). Based on the obtained data it was observed that the relative viscosity of the silane compounds solution prepared in toluene initially rises with increasing concentration (maximum for FDTS achieved at 0.05% and for FPTS at 0.10%) and next decreases with increasing content of perfluoroalkylsilane compounds, while for surface tension measurements a reversed behavior was observed. It was noticed that the minimum values of this parameter corresponded to the maximum values of relative viscosity.

These results are related to the appearance of critical concentration and interfacial tension [38,39]. Furthermore, the authors found that this activity was higher for smaller concentrations of linear fluorosurfactants, which affect the decrease in surface tension. A similar tendency for perfluoroalkylsilanes was confirmed in our measurements. In addition, the strong influence of van der Waals and electrostatic interactions between individual molecules was observed, which as a consequence influenced the lowest indications of surface tension and higher values of relative viscosity. In our studies, this phenomenon was observed for the solutions of perfluoroalkylsilanes with a concentration of 0.05% for FDTS and 0.10% for FPTS (indicating their higher activity), respectively.

**Figure 2:**
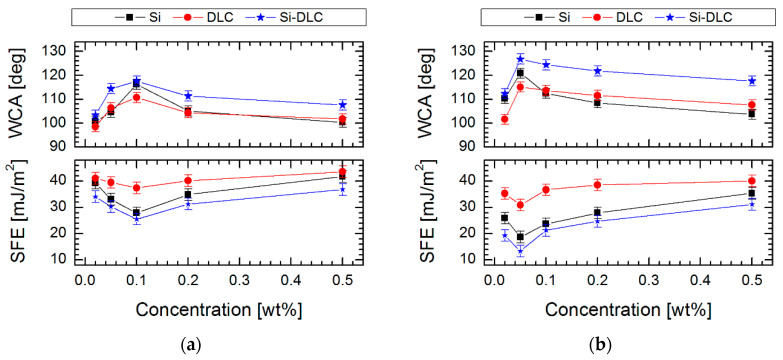
T Water contact angle (WCA) and surface free energy (SFE) of self-assembled monolayers (SAMs) created on three surfaces with different contents of silicon as function a concentration for (**a**) FPTS and (**b**) FDTS.

In Figure 2 are presented the water contact angle (WCA) and surface free energy (SFE) of SAMs created on DLC, Si-DLC and Si substrates using the above-described perfluoroalkylsilanes solutions. For the investigated substrate materials, the highest WCA was obtained after FDTS modification with 0.05% concentration, (126.8 ± 2.0°) for Si-DLC coating, (120.9 ± 2.0°) for Si surface and (115.1 ± 2.0°) for DLC coating, while achieving a low value of SFE. The results described above allow the conclusion that the alterations of wettability can be connected with the fluctuations of surface tension and viscosity of the solution used. The highest values of WCA occurring at the solution concentrations used to form SAMs revealed the highest relative viscosity and the lowest surface tension (Figure 1). For SAMs obtained using FDTS, these values were observed at 1.35 of viscosity and 22.50 mN/m of surface tension of the used solution. For comparison, FPTS layers revealed the most promising values of WCA (117.6 ± 2.0° for Si-DLC) at 1.24 of viscosity and 27.11 mN/m of surface tension of the solution. Additionally, a critical concentration value, above which there were no significant changes in water contact angle, viscosity and surface tension measurements, was observed. This effect indicates the changes of van der Waals and electrostatic interactions between perfluoroalkylsilane molecules and promotes the formation of micelles or micelle-like aggregates. It was found that the same effect is also observed for surfactants [38].

### 3.2. FTIR Spectroscopy Analysis of Investigated Surfaces

**Figure 3:**
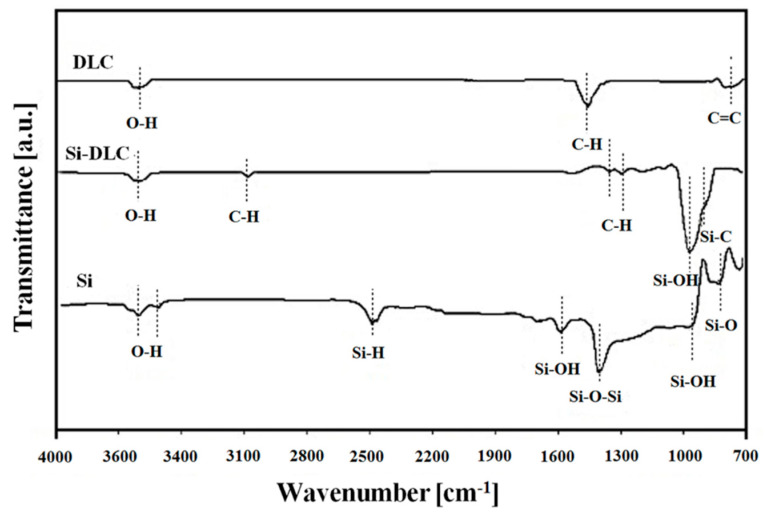
Fourier transformed infrared spectroscopy (FTIR) comparative study of diamond-like carbon (DLC), silicon-incorporated DLC (Si-DLC) and Si surfaces.

The substrates with different silicon concentration, 0% (DLC), 32% (Si-DLC) and 100% (Si wafer), were characterized using a FTIR spectrometer (Figure 3). The presented results show differences in the amount and intensity of hydroxyl groups obtained between 3500 and 3700 cm^−1^. On the FTIR spectrum for Si-DLC, C–H vibrations at 1300 cm^−1^, 1387 cm^−1^ and 3105 cm^−1^ are visible. This indicates that Si-DLC contains more hydrogen in its matrix than non-incorporated DLC coating. The role of hydrogen in the physical-chemical properties of diamond-like carbon coatings has been confirmed by Erdemir [40]. However, the influence of hydrogen on these materials will be described in the section related to the tribological behavior. Moreover, the occurrence of a signal at 938 cm^−1^ assigned to the Si-OH bond and a signal at 864 cm^−1^ assigned to the Si–C bond was observed. This confirms that both silicon and oxygen are incorporated in the structure of Si-DLC coating.

**Figure 4:**
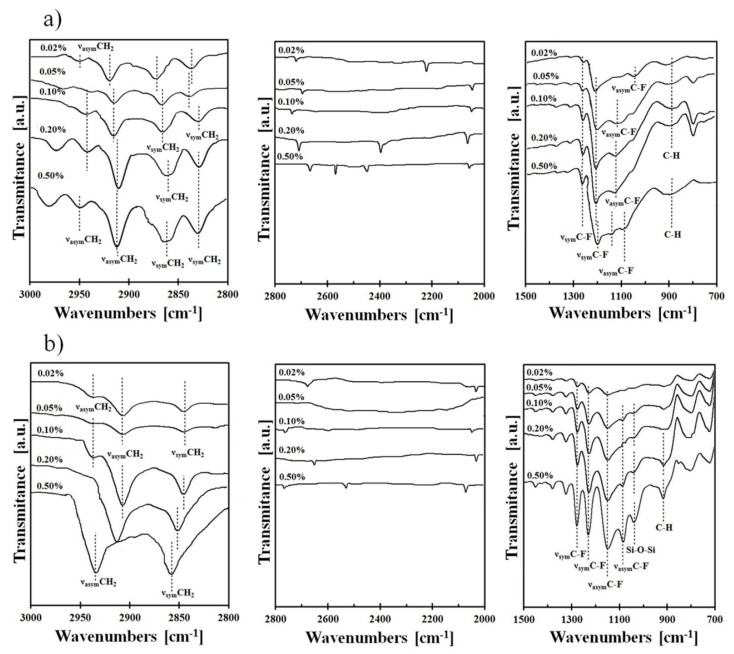
FTIR of typical spectra for (**a**) Si-DLC/FPTS and (**b**) Si-DLC/FDTS in the ranges of 1500–700 cm^−1^, 2800–2000 cm^−1^ and 3000–2800 cm^−1^.

FTIR spectroscopy was also used for the characterization of the surface after chemical modification. It was found that the most interesting region for observing the fluoroalkyl layer structure due to the major changes in spectra was from 700 to 1500 cm^−1^ and in the region 2000–3000 cm^−1^. The typical spectra in these regions for the Si-DLC coating modified using FPTS and FDTS in five different concentrations (0.02–0.50%) are presented in Figure 4.

**Figure 5:**
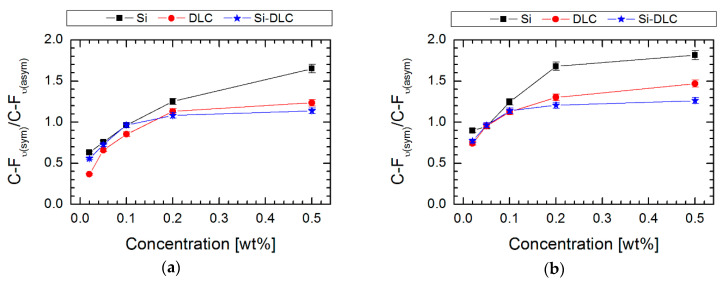
The area ratio of FTIR bands assigned to symmetric and asymmetric C-F stretching vibration versus concentration of perfluoroalkylsilanes used during the liquid phase deposition (LPD) process for (**a**) FPTS and (**b**) FDTS compounds.

**Figure 6:**
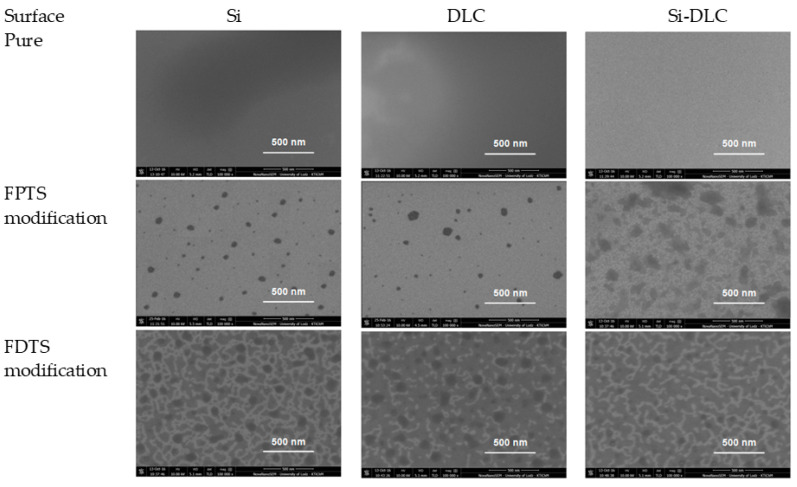
The scanning electron microscopy (SEM) images of three different surfaces after creation of FPTS and FDTS SAMs.

Absorption maxima in the range of 950–1100 cm^−1^ are characteristic for Si–O–Si stretching vibrations, which indicate horizontal siloxane connections and promote the formation of covalently attached monolayers, leading to a self-organization process [41]. It is also worth noting that the obtained maxima observed at about 900 cm^−1^ are associated with a deformation of vibrations obtained for C–H stretches originating from –CH_2_–CH_2_–, a fragment of the alkyl chain. Nevertheless, the most essential peaks for our investigations (identified in the range 1100–1350 cm^−1^) were revealed between carbon and fluorine, which correspond to asymmetric and symmetric stretches. It was established that these observations are clear evidence of the modification process. The intensity of C–F peaks increases with the concentration of perfluoroalkylsilane used for deposition of SAMs on three surfaces. The areas of these peaks (symmetric at 1250–1300 cm^−1^ and asymmetric at 1100–1200 cm^−1^) were integrated. Based on these data, the ratio of symmetric to asymmetric vibrations of C-F bonds was calculated. In every case (Figure 5), the ratio increases with higher saturation of the investigated solution. It means that the degree of coverage of the investigated surfaces increases. It was found that the most appropriate coverage by compounds is when the ratio of investigated areas is close to one [42]. Comparing this fact with the measurements of the contact angle (Figure 2), we can conclude that in this case, the obtained layers show the highest hydrophobicity. Confirmation of the high coverage ratio of the analyzed surfaces using SAMs is visible on the SEM image of the surface with the highest hydrophobicity (Figure 6). EDS maps of those areas indicate ~10 wt% of fluorine next to atoms characteristic for modified coatings/surfaces.

Another factor determining the physical-chemical properties of the manufactured layers is their thickness. Here, FTIR spectroscopy is one of the techniques used for determination of the thickness of the prepared layers [43,44,45]. These measurements were obtained based on the formula:

(1) $$h=\frac{1}{2}nN/\left(\upsilon_{1}-\upsilon_{2}\right),$$

where, *h*—layers thickness; *n*—refractive index of the investigated surface/layer; *N*—number of fringes within a spectral region and *υ*_1_, *υ*_2_—starting and ending points in the spectrum in cm^−1^.

A condition of determining the thickness of manufactured layers from FTIR investigations based on Equation (1) is the presence of infrared interference [46], which occurs as a “fringing effect” [47]. In our studies, this effect had been observed between 2000 and 2800 cm^−1^ (Figure 4). In this region, the starting and ending points and the number of fringes were determined. The results of the calculations of thickness are shown in Table 1.

**Figure 7:**
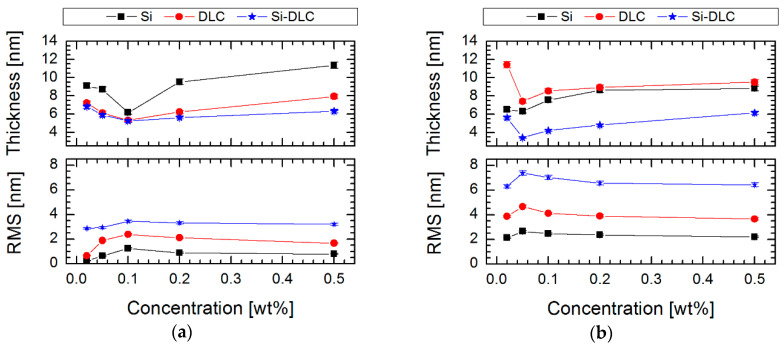
Effect of concentration solution of perfluoroalkylsilane on thickness and roughness (RMS) of SAMs created on three surfaces with different contents of silicon for modifiers: (**a**) FPTS and (**b**) FDTS.

To confirm these results, a spectroscopic ellipsometry technique was used for thickness measurements (Figure 7). The obtained results indicate a thin layer of SAMs, especially for Si-DLC/0.05% FDTS and Si-DLC/0.1% FPTS systems. This behavior can be explained by the differences in the structure and the tendency in orientation relative to the investigated surface. It means that shorter compounds like FPTS, with the length obtained from the theoretical model, determined using HyperChem 7.5 equal to 0.70 nm, tend to form a vertical orientation, which is connected with the creation of a multi-layer structure (thickness from 8.81 nm for 0.50% to 4.46 nm for 0.10%). On the other hand, longer compounds like FDTS, with a theoretical length equal to 1.64 nm [48], create horizontally oriented structures relative to the investigated surface and, as a consequence, better packed structured coverage. Thus, it can be stated that both the type of the modifier and the solution concentration has a significant impact on the thickness of the layer, as can be seen in Figure 7.

Further analysis of FTIR spectra in the region 2800–3000 cm^−1^ (Figure 4) shows differences in C–H symmetric and asymmetric stretching vibrations that originated from the hydrocarbon part of the silane chain. It was found that these changes depend on the occurrence of ordered (crystalline-like) or disordered (liquid-like) structures [49]. The lack of asymmetric C–H bonds or the occurrence of these stretches with low intensity, like in the case of Si-DLC/0.05% FDTS, indicates a crystalline-like structure. This structure designates well-packed layers with good coverage, which showed two low intensity asymmetric stretching modes visible at 2939 cm^−1^ and 2921 cm^−1^, whereas the liquid-like structure is manifested by one or more absorption bands with high intensities and/or vibrations shifted to higher wavenumbers. Hence, a liquid-like structure that occurs on defects due to disorder was observed for a system composed of Si-DLC and 0.50% FDTS (one peak with high intensity at 2943 cm^−1^). Therefore, the presence, position and intensity of these bonds decides the ordering of the manufactured structures.

### 3.3. Tribological Properties

**Figure 8:**
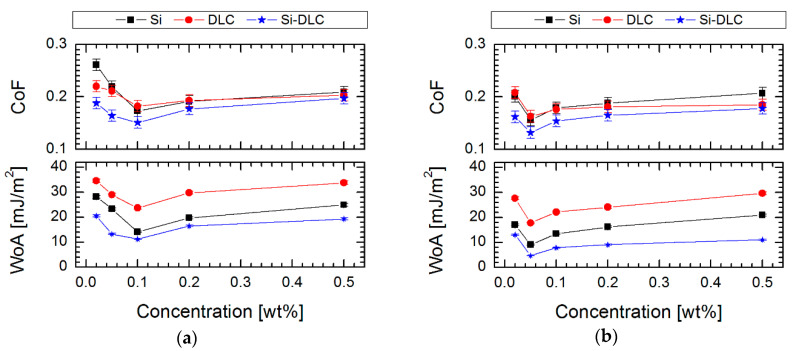
Coefficient of friction (CoF) and work of adhesion (WoA) of SAMs created using the LPD process on three surfaces with different contents of silicon as a function of the concentration of solutions of (**a**) FPTS and (**b**) FDTS compounds.

The tribological properties at millinewton load (values of friction coefficient) for all investigated surfaces are shown in Figure 8. It was found that the Si-DLC coating showed better friction properties in comparison to the DLC and silicon surface (CoF = 0.21, 0.24 and 0.31 respectively). This trend is visible not only for unmodified surfaces but also after the modification. The changes in these properties between the analyzed surfaces are linked with the following factors: (i) a presence of hydrogen, (ii) a presence of native silicon oxide as well, (iii) wettability of the surface and (iv) thickness of deposited layers.

The differences in the hydrogen content of DLC and Si-DLC coatings and the Si surface were evaluated by analyzing the signals of C-H bonds (Figure 3). In the case of the Si-DLC coating, in the FTIR spectra three low intensity bands assigned to C-H vibrations are observed, whereas for pure DLC only one strong band is visible. The area ratios of bands assigned to C–H vibrations and to other bands that occur in the spectra were calculated: 14% for Si-DLC and 28% for DLC coating. These results allow the assumption that the relative content of hydrogen in DLC coating is two times higher than in Si-DLC coating. Erdemir [40] indicate that highly hydrogenated carbon coatings during a tribological test performed in the open air absorbed more oxygen species and water molecules than other surfaces. Therefore, one of the reasons for the occurrence of better microtribological performance obtained by the lower coefficient of friction for Si-DLC coating (0.214 ± 0.011) compared to DLC coating (0.240 ± 0.011) is linked with the presence of native silicon forms (SiO_x_) [50]. Silicon oxides in combination with the presence of water in the form of water meniscuses enhance the formation of a Si(OH)_4_ layer at the top of the investigated surfaces according to the following reaction:

(2) $${\mathrm{SiO}}_{2}+{2H}_{2}O\to \mathrm{Si}{\left(\mathrm{OH}\right)}_{4},$$

The appearance of SiO_x_ and Si(OH)_4_ in this study is confirmed by the presence of Si–O and Si–OH signals in FTIR spectra. Figure 3 shows the differences in the presence of these vibrations between Si substrate and Si-DLC coating. It is seen that in the case of the silicon-incorporated diamond-like carbon coating one peak characteristic for Si–OH with significant intensity is visible. It was established by Bhushan that the amount of silicon oxides as well as the presence of a Si(OH)_4_ layer is one of the main reasons determining microtribological behavior [51]. Therefore, in the case of silicon-incorporated coating, presence of SiO_x_ promotes the formation of a thin layer containing Si(OH)_4_ particles in a carbon matrix, which reduces friction in comparison to DLC coating. However, the FTIR analysis showed that the Si(OH)_4_ bonds occur not only for Si-DLC coating but also for the Si surface. Results of microtribological tests showed that despite the presence of silicon oxides, silicon was the substrate with the highest value of friction coefficient (0.308 ± 0.011). This indicates that a form of presence of Si(OH)_4_ in surface layers determines their tribological properties. It appears the continuous monolayer of silicon oxide on the top of the silicon results in a high friction coefficient, whereas Si(OH)_4_ particles distributed in carbon matrix composed of C-H bonds (the case of Si-DLC coating) ensures a low friction coefficient.

From the above-mentioned factors, the wettability of the surface has a significant impact on the microscale tribological behavior. If we look at the previously presented results of the wettability (Figure 2), it was found that the silicon surface has the lowest contact angle compared to Si-DLC and DLC. This is related to the hydrophilic character of the silicon, which can easily adsorb water from the environment. As is well known, the water has a negative influence on the friction, because between the counterbody and the surface, adhesive connections are formed which are difficult to break. 

In the case of modifications, it was supposed that the amount of silicon is equally important in the context of the effect of deposition of silane SAMs. It was found that silicon plays a role of anchoring center, which enhances the formation and ordering of silane structures. The tests in the millinewton load range exposed improving tribological properties by lowering the coefficient of friction after modification up to 0.132 ± 0.011 for Si-DLC/0.05% FDTS system. It proves that thin perfluoroalkylsilane layers can provide good lubrication between Si_3_N_4_ ball and hydrophobic surfaces. It was also demonstrated that the less effective lubrication was obtained on more hydrophilic surfaces, which indicated lower RMS values (Figure 7) than in the case of the most hydrophobic systems (Figure 2). A possible increase of roughness after modification for less hydrophobic layers is caused by local aggregation of modifier compounds [51], which also decides the increasing thickness for these coatings and influences the values of the coefficient of friction (Figure 8). Moreover, it was found that the hydrophilic character of the modified systems favors partial forming of water meniscuses on the top of these surfaces. Hence, in the case of tribological measurements, where the ball loaded in the millinewton load range is in contact with a flat surface, the presence of attractive force is caused by meniscus force. This attractive force is called Laplace force (F_L_) and is expressed by the following equitation:

(3) $$F_{L}=2\pi R\gamma_{LV}\left(\cos\theta_{1}+\cos\theta_{2}\right),$$

where, *R* is the radius of the water sphere; *γ_LV_* is the surface tension of the liquid against air; *θ*_1_ and *θ*_2_ are the contact angles between liquid and surfaces [51,52,53].

Furthermore, it was established that Si_3_N_4_ balls have a considerable radius, hence the Laplace force and van der Waals impact is large. Therefore, meniscus force is the main factor determining adhesion in the presented investigations. Influencing these forces leads to alterations of the work of adhesion. The work of adhesion was calculated based on Equation (4). This equation introduced a quantity on real surfaces of the apparent contact angle of static water for a water drop of a solid surface using the Young-Dupre equation [51]:

(4) $$W_{e}=\gamma_{LV}\left(1+\theta_{e}\right),$$

where, *θ_e_* is the equilibrium (Young’s) contact angle.

The performed investigation (Figure 8) indicated a major reduction of the work of adhesion for systems modified using perfluoroalkylsilane in comparison to pure materials (67, 120 and 104 mJ/m^2^ respectively for DLC, Si and Si-DLC), which confirms the effectiveness of the performed modifications. In the case of modification, it was also found that solution concentration has a significant effect on the quality of the obtained monolayer and, as a consequence, on the values of adhesion work. Moreover, Liu and Bhushan [51] indicated the relationship between the structure chemistry of produced SAMs and their adhesive properties. It was presented that with higher hydrophobicity, the impact of van der Walls force is smaller on the adhesive force. As a consequence, for hydrophobic surfaces, adhesive force is proportional to the work of adhesion [51,52,53]. In our measurements the work of adhesion confirms the authenticity of the described phenomenon. Taking into account the concentration of perfluoroalkylsilanes, where modification is the most effective, the work of adhesion was 23.86, 14.26 and 11.75 mJ/m^2^ for FPTS and 17.79, 9.10 and 5.37 mJ/m^2^ for FDTS SAMs created on DLC, Si and Si-DLC substrate materials, respectively (see in Figure 8). Comparing the type of modifier, the lowest value was obtained for the system composed of Si-DLC coating and 0.05% FDTS. These studies suggest that FDTS films can perform as thin, well-ordered, anti-adhesion films with good coverage, which significantly reduces the work of adhesion.

**Figure 9:**
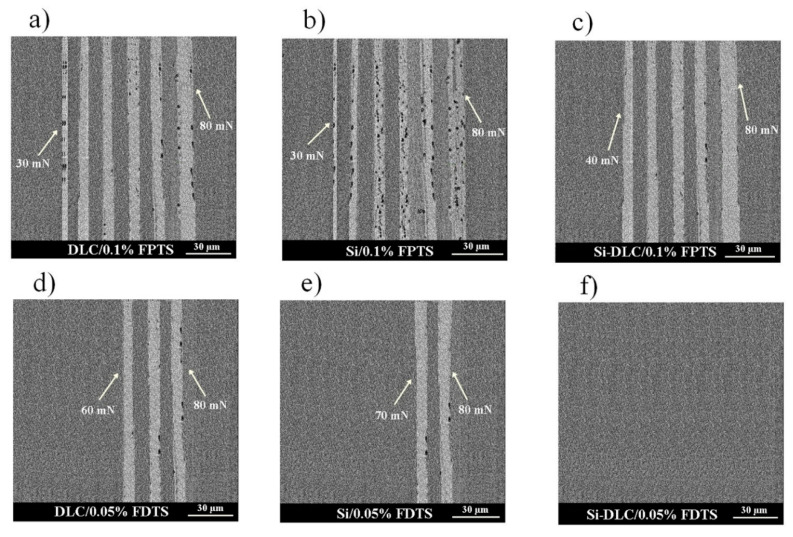
The SEM images of the surface of investigated layer structures after tribological tests: (**a**) DLC/0.1% FPTS, (**b**) Si/0.1% FPTS, (**c**) Si/0.1% FPTS, (**d**) DLC/0.05% FDTS, (**e**) Si/0.05% FDTS, (**f**) Si-DLC/0.05% FDTS.

The last parameter influencing the friction is the thickness of SAMs. Generally, high friction is caused by thick and non-ordered layers with visible traces of wear after microtribological tests, especially for FPTS modification. For this perfluoroalkylsilane modification at the concentration of 0.1% for the whole range of load and for all studied surfaces, wear tracks were registered (Figure 9). Wear track analysis also shows that Si-DLC/0.05% FDTS presented better microtribological properties in comparison to other modifications due to the lowest coefficient of friction and lack of wear after friction tests. This fact is due to the previously discussed occurrence of ordered (crystalline-like) or disordered (liquid-like) structures as well as layer thicknesses of 4.46 nm and 3.29 nm for FPTS and FDTS respectively.

## 4. Conclusions

Two homolog perfluoroalkylsilanes (FPTS and FDTS) were used to form self-assembled monolayers on DLC, Si-DLC and silicon substrates via the liquid phase deposition technique. The different concentrations of perfluoroalkylsilanes solution used to form SAMs allow indication of the system characterized by the best coverage of the surface with the highest hydrophobic properties. Comparing these two modifications, it was found that FDTS, due to the tendency of vertical polymerization and more fluorine in the structure, allows manufacturing of thinner, well-covered and more crystalline-like layers with higher WCA than in the case of FPTS modification. Moreover, results of the coefficient of friction, work of adhesion and wear investigations are in a close relationship and confirm the effectiveness of modification using a 0.05% concentration of FDTS in toluene. The investigation also enabled the best material candidate to be chosen as a substrate for the liquid perfluoroalkylsilane modification process. It was found that the DLC coating containing silicon is more attractive than silicon wafer and pure DLC coating. This is caused by the presence in DLC coating of dispersed native silicon forms (SiO_x_) which constitute active centers for chemical bonds and as a consequence improved chemical modification.

Concluding, the low values of adhesion and coefficient of friction as well as the obtained high hydrophobicity registered, especially for Si-DLC modified using FDTS with a concentration of 0.05% (126.8 ± 2.0°), makes these systems attractive, especially for MEMS devices, where intensive tribochemical processes take place.

## Figures and Tables

**Table 1:** Thickness [nm] of SAMs created on different substrates using LPD method using the solutions of FPTS and FDTS compounds at five different concentrations, estimated in FTIR measurements. Relative uncertainty is lower than 2%.

Substrate Material	FPTS Concentration					FDTS Concentration				
	0.02%	0.05%	0.10%	0.20%	0.50%	0.02%	0.05%	0.10%	0.20%	0.50%
DLC	7.15	6.03	5.44	6.50	12.68	11.22	7.64	9.04	9.60	9.27
Si-DLC	6.63	5.07	4.46	5.18	8.81	5.23	3.29	4.07	4.70	6.21
Si	8.76	8.65	5.09	9.89	16.45	6.61	5.81	7.39	8.82	7.97
